# The Relationship of Urban Form on Children and Adolescent Health Outcomes: A Scoping Review of Canadian Evidence

**DOI:** 10.3390/ijerph18084180

**Published:** 2021-04-15

**Authors:** Tona M. Pitt, Janet Aucoin, Tate HubkaRao, Suzanne Goopy, Jason Cabaj, Brent Hagel, Gavin R. McCormack

**Affiliations:** 1 Department of Paediatrics, Cumming School of Medicine, University of Calgary, 28 Oki Drive NW, Calgary, AB T3B 6A8, Canada; tate.hubkarao@ucalgary.ca (T.H.); brent.hagel@albertahealthservices.ca (B.H.); 2 Department of Community Health Sciences, Cumming School of Medicine, University of Calgary, 3280 Hospital Drive NW, Calgary, AB T2N 4Z6, Canada; janet.aucoin1@ucalgary.ca (J.A.); jason.cabaj@albertahealthservices.ca (J.C.); gavin.mccormack@ucalgary.ca (G.R.M.); 3 Faculty of Nursing, University of Calgary, 2500 University Drive NW, Calgary, AB T2N 1N4, Canada; sgoopy@ucalgary.ca; 4 Usher Institute, Old Medical School, University of Edinburgh, Teviot Place, Edinburgh EH8 9AG, Scotland, UK; 5 Alberta Health Services, 10301 Southport Lane SW, Calgary, AB T2W 1S7, Canada; 6 Alberta Children’s Hospital Research Institute, University of Calgary, 28 Oki Drive NW, Calgary, AB T3B 6A8, Canada; 7 Sport Injury Prevention Research Centre, Faculty of Kinesiology, University of Calgary, 2500 University Drive NW, Calgary, AB T2N 1N4, Canada; 8 O’Brien Institute for Public Health, University of Calgary, 3280 Hospital Drive NW, Calgary, AB T2N 4Z6, Canada; 9 School of Architecture, Planning and Landscape, University of Calgary, 2500 University Drive NW, Calgary, AB T2N 1N4, Canada; 10 Faculty of Kinesiology, University of Calgary, 2500 University Drive NW, Calgary, AB T2N 1N4, Canada; 11 Faculty of Sport Sciences, Waseda University, 1-104 Totsukamachi, Shinjuku-ku, Tokyo 169-8050, Japan

**Keywords:** urban form, child, youth, adolescent, food environment, health, injury, obesity, built environment

## Abstract

Urban form can have an impact on health outcomes in children, and the synthesis of findings can identify gaps in the literature and regional reviews may help guide policymakers. This study aims to complete a scoping review of the research relating urban form to health outcomes in children and adolescents from urban Canadian settings. Thirteen online databases were searched to identify studies that had objective measures of urban form and health outcomes. Two research assistants independently reviewed 27,444 titles and abstracts, and 176 full-text articles, returning 32 unique studies with youth-specific data. The majority of the included studies were cross-sectional or ecological (*n* = 26). Six studies used Canada-wide data and the rest were from Ontario (*n* = 11), Alberta (*n* = 6), and Quebec (*n* = 6). Urban form characteristics included neighbourhood food environment (*n* = 11), parks/natural space/greenness (*n* = 10), road or intersection characteristics (*n* = 7), and aggregated urban form measures (*n* = 7). Studies examined a variety of health outcomes: the majority considered weight status (*n* = 16) and injury (*n* = 10). Although there is over-reliance on mainly cross-sectional study designs, there is evidence suggesting that urban form is associated with health outcomes in Canadian youth, with parks/greenspace, road connectivity, and road characteristics most consistently associated with health outcomes in youth.

## 1. Introduction

Population-level interventions are needed to reduce the incidence of lifestyle-related diseases that significantly burden provincial healthcare systems [1]. Creating a health-supportive urban form, a population-level intervention, has been identified as an important strategy for reducing chronic disease risk and promoting healthy behaviours in Canada and globally [2,3]. A previous scoping review explored the relationship between health outcomes and urban form (referring to “physical surroundings and features such as parks, streets, buildings, destinations and land uses, connectivity, density, sidewalks and paths, lighting, aesthetics, and architecture” [4]) specific to research undertaken in Canada [4] and found consistent associations between transportation routes/connectivity and injury as well as aggregate neighbourhood measures of walkability and weight status [4]. The review, however, was not generalizable to the entire population as it was restricted to adults. This is significant as urban form may influence behaviours and health outcomes differently in children and adolescents insofar as their primary use of the urban form may differ (i.e., for recreation rather than utilitarian). Furthermore, there is evidence that health behaviours (e.g., physical activity) and risk factors for health conditions (e.g., overweight, diabetes, metabolic syndrome) track from childhood into adulthood [5,6,7]. Poor health in children and youth may lead to poor health in adulthood, and thus it is important to consider interventions that serve as primary prevention tools: younger populations and their interactions with the urban form should be targeted to improve health early in life.

When guiding decision making and future directions related to land use and environments that are health-supportive, especially in Canada, reviews provide a resource to synthesize impactful findings [8]. Previous systematic reviews on urban form and health, however, typically do not stratify their findings by country or geographical region and, despite being informative, lack specificity, feasibility, and relevance for aiding local (Canadian) decision making [4]. Previous work has indicated differences exist between countries with regard to physical activity and health outcomes. For example, differences in cycling levels exist between the United States and Canada [9], which might be explained, in part, by differences in urban form, costs of vehicle ownership, income levels, and cycling experience between the two countries [9]. Other studies have found cross-national differences in associations between socioeconomic status and youth obesity attributed to differences in “social, economic, and environmental factors” across countries [10]. Researchers have also found country differences in youth obesity attributable to socio-demographic and political variation, macro-economic indicators, federal public health spending, number of motor vehicles, and overall health status across European countries [11]. The climatic, cultural, political, legislative, and healthcare system differences between countries suggest that synthesizing findings from studies undertaken within a single country is a potentially useful strategy for informing local urban planning strategies and policies that have the ability to improve health [4,11,12]. Indeed, existing systematic reviews on urban form and health in children and youth are often heavily weighted towards particular countries (e.g., USA [13]) or contain little evidence specifically from Canada [14]. Thus, evidence from such reviews does not always offer evidence that is generalizable to Canada or constituent provinces.

The influence of specific built environment changes may affect health outcomes within particular countries differently due to the inherent social norms and culture; that is, all things being equal, a change in urban form might result in different health behaviours and outcomes depending on the context of that particular country. In children, cross-cultural differences and their subsequent effect on active mobility are more pronounced given that “the impact of the built environment is influenced by the decision making of parents as the gatekeepers of their behaviour” [15]. Caregiver views and social/cultural norms can have as significant an influence on active school transportation as the urban form (i.e., building sidewalks) [16]. Variations in the perception of safety across countries and regions [17], alongside national policies and norms such as cost for children to bus to school, walking school bus funding, and working parents, can differ across countries and influence child active transportation [18]. Irregularities brought about by national policies, cross-cultural practices, and social norms suggest that even if the same intervention and environment were implemented across countries, the magnitude (and perhaps direction) of effect may differ considerably due to regional differences not captured solely by the urban form. In fact, there may even be regional differences within a single country; however, when data reviewed pertain to a single country, it is possible for information to be organized with due consideration of political boundaries (i.e., provinces).

While there has been recent work summarizing evidence related to health outcomes associated with urban form in adults in the Canadian context [4,19], there is no single resource that specifically addresses these associations in children and youth. Using a scoping review strategy, we summarized studies investigating the associations between urban form and health outcomes in children and adolescents in Canada. From this review, we sought to better understand the methods and findings of research being undertaken in Canada on this topic, specifically what health outcomes are being studied in relation to urban form, what urban form characteristics have been of focus to date, the strategies used to objectively measure urban form, and the geographical locations of where, and the populations within which, studies have been conducted. Through our review, we identify knowledge gaps and propose potential future directions for research with a focus on how policy and placemaking could affect the health of children in Canada. This scoping review [20] strategy allows flexibility to map current knowledge related to relatively broad research questions, in contrast to a systematic review that is limited to a particular, often specific, question. Importantly, this scoping review will be useful for informing the development of specific research questions related to urban form and health outcomes in Canadian children and adolescents.

## 2. Materials and Methods

### 2.1. Overview

To facilitate greater reproducibility and transparency, this review follows the method proposed by Arksey and O’Malley [20] as well as the PRISMA scoping review guidelines [21]. This review, focussed on children and adolescents, complements a previous scoping review, which was undertaken to better understand the associations between urban form and chronic health conditions among adults in Canada [4].

### 2.2. Inclusion Criteria

Inclusion criteria were guided by our previous research but restricted to children and youth [4]. Studies were considered eligible if they included all of the following: a sample recruited from a Canadian population; a focus on the youth population (≤18 years of age); an objective measure of urban form (exposure); and a measure of health outcomes. The objective measure of urban form, for example, might include the density of convenience stores within a 500 m radius of a child’s home. This is in contrast to subjective measures whereby parents or children might report perceptions regarding the walkability of their neighbourhood. Thus, our review only included quantitative studies or mixed method studies with a quantitative component. The focus of the review was on health outcomes, but not health behaviours. For instance, studies that exclusively examined active school transportation, physical activity, sedentary behaviour, diet, smoking, alcohol, or other drug use but did not include health outcomes (e.g., disease, injury, well-being, mortality) were excluded. Studies where there was no direct physical interaction between urban form characteristics and the participant were excluded. For example, studies exclusively investigating pollution, electromagnetism, and traffic noise, or where urban form was explicitly used as a proxy for pollution, were excluded. We also restricted our review to studies undertaken in urban settings and excluded studies that only sought to compare urban versus rural areas. Studies with samples that included both youth and adult participants remained in the review if they estimated separate associations between urban form and health among youth.

### 2.3. Search Strategy

Our scoping review included peer-reviewed articles only and excluded other types of publication (dissertations, conference abstracts, commentaries, and reviews). We examined reference lists of relevant reviews; however, this did not result in the inclusion of any articles not already captured in the initial search. There were no restrictions on article language. The search strategy did not restrict articles based on their publication date and included articles from all available years.

Our scoping review included a search of thirteen online databases (CINAHL, EMBASE, Environment Complete, MEDLINE, PsycInfo, PubMed, Scopus, SocIndex, SportDiscus, TRID, Urban Studies, Web of Science, and CAB Abstracts). The search strategy and databases used were guided by a librarian scientist (University of Calgary) and informed by a previous scoping review [4]. The search was completed in February 2021. Separate title, abstract, and keywords searches were conducted for the urban form (*n* = 58), Canadian geography (*n* = 46), and population-specific terms (*n* = 19) (MEDLINE search strategy in Figure S1). We combined the three searches to identify relevant studies. A subsample of abstracts (*n* = 3000) was selected to evaluate consistency in applying the inclusion and exclusion criteria by the two research assistants (inter-rater reliability: kappa = 0.73; 95% CI: 0.63–0.83 and percent of overall agreement = 99.1%). Similarly, the two research assistants applied the inclusion and exclusion criteria to selection of the full-text articles (inter-rater reliability: kappa = 0.57; 95% CI: 0.42–0.71 and percent agreement = 81.4%). Where disagreement regarding abstract or full-text article inclusion or exclusion existed, the research assistants reached consensus with guidance from the other authors.

### 2.4. Synthesis

Two research assistants (T.P. and J.A.) developed the data chart, based on a previous scoping review in Canada [4], for the full-text evaluation and data extraction. The data charting included information on the journal, year of publication, year(s) studied, author information, city/province/country of study, study design, peer review status, health outcome(s), urban form characteristic(s), data source, sample size, statistical estimate of association or statistical comparison, covariates included in the model, and main outcomes. Studies were grouped by urban form characteristic(s) informed by an existing framework on the urban form [18]. In the context of social determinants of health and health promotion, this framework emphasizes the modifiability of urban form categories (e.g., land use, transportation systems, and public resources). Briefly, the framework includes four levels; the first consists of natural environment, macrosocial, and inequalities. These fundamental factors influence the next intermediate levels of built environment and social context, with emphasis on the built environment (the built-up area existing within the urban form). These intermediate factors, in turn, affect proximal factors such as stressors (e.g., violent crime and financial insecurity), health behaviours, and social integration/social support. Finally, these proximal factors influence health and well-being; this category is subdivided into health outcomes and well-being. Health outcomes related to the urban form include the categories of infant and child health, obesity, cardiovascular diseases, diabetes, cancers, injuries and violence, infectious disease, respiratory health, mental health, and all-cause mortality [22]. These categories for urban form and health outcomes were used to inform the way in which articles included in the review were categorized.

## 3. Results

### 3.1. Overview of Included Studies

**Figure 1:**
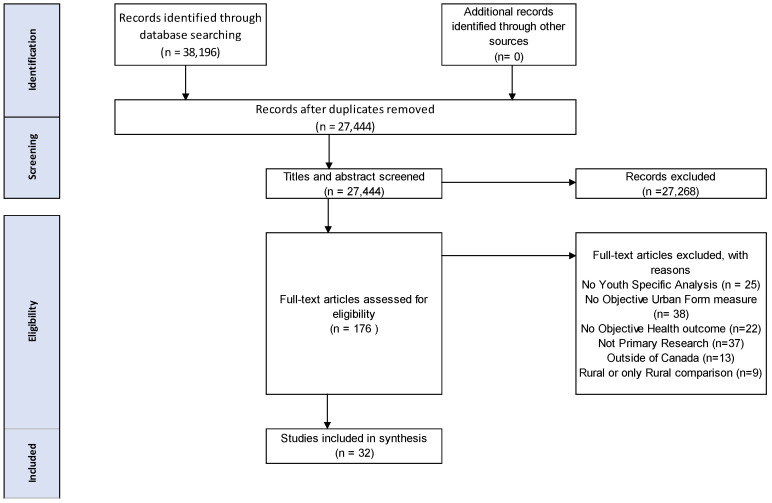
PRISMA-ScR flow chart of included and excluded studies.

The search returned 38,196 titles, and after de-duplication, there were 27,444 unique titles and abstracts for review (Figure 1). After abstract screening, 176 titles were considered eligible for full-text review. After applying the inclusion and exclusion criteria, we extracted data from 32 full-text (31 English and 1 French language) articles estimating associations between urban form and health outcomes among children (Table S1). A brief overview of frequency by category is provided in Table 1.

Articles included in this review were published between 1992 and 2019. Despite this publication date range, the data included in these articles were collected between 1978 and 2017. Of these studies, however, 25 were published after 2010 and only one was published before the year 2000. Studies were published in multiple disciplines with a focus on public health and medicine (e.g., International Journal of Obesity, AIMS Public Health, Public Health Nutrition, Canadian Journal of Public Health, International Journal of Public Health, BMC Public Health), injury (e.g., Injury Prevention, Accident Analysis & Prevention, Injury Epidemiology, Traffic Injury Prevention), and environmental health (e.g., International Journal of Environmental Research and Public Health). The majority of studies were cross-sectional or ecological (*n* = 26), and the remaining studies were case–control (*n* = 3) or longitudinal (*n* = 3). Six studies used Canada-wide (population-level) data (Canadian Community Health Survey [23], Canadian Health Measures Survey [24], and Health Behaviour in School-Aged Children Survey [25,26,27,28]). The remaining articles were geographically specific, including data collected in Ontario (*n* = 11), Alberta (*n* = 6), Quebec (*n* = 6), British Columbia (*n* = 2), Saskatchewan (*n* = 1), and Manitoba (*n* = 1) (note that some studies may examine multiple provinces). There were no other provinces or territories represented with geographically specific results. Only five studies used the entire age range of under 19 years [28,29,30,31,32,33]. Other studies included specific age groups (e.g., 8–10 years of age [34,35,36,37], under 14 years [38], over 14 years [39]) or school grade groups (e.g., Grades 6–10 [40,41,42], Grades 5–6 [43], Grades 1–4 [44], and Grades 5–8 [45]). Some studies used the same cohort data from the Quebec Adipose and Lifestyle Investigation in Youth (QUALITY) cohort [28,34,35,36]; this cohort was the only study using specific individual characteristic inclusion criteria in addition to age: Caucasian students with at least one obese parent. Sample size for studies ranged from as few as 33 [30] to as many as 326,383 children and adolescents [41].

Studies examined a variety of health outcomes, including weight status (obesity/body mass index/adiposity; *n* = 16), injury (*n* = 10), mental health (*n* = 2), asthma/respiratory illness (*n* = 1), mortality (*n* = 1), allergic rhinitis (*n* = 1), and health-related quality of life (*n* = 1). Urban form characteristics investigated included parks, natural space and greenness (*n* = 10), road and intersection characteristics (*n* = 7), aggregate measures of neighbourhood (i.e., walkability, measure of sprawl, and neighbourhood design) (*n* = 7), residential neighbourhood food environment (*n* = 6), school neighbourhood food environment (*n* = 5), access to recreation opportunities (i.e., sports facilities, playgrounds, etc.) (*n* = 4), street connectivity (*n* = 3), population/dwelling density (*n* = 2), and road density (*n* = 1). Studies often included multiple urban form characteristics simultaneously (e.g., food environment and walkability). Frequency of urban form and health outcomes were mapped (Table 1).

### 3.2. Measurement of Urban Form

Urban form was measured most frequently using geographic information systems (GIS) (*n* = 20). The analyses using GIS primarily included municipal data (*n* = 10) and DMTI license databases such as CanMap and Enhanced Points of Interest (*n* = 9). Some studies also conducted in-person environmental audits [34,46,47], virtual confirmation using Google Earth [46], or confirmation through business and telephone directories [23,37,48]. Two [34,47] of the in-person audits were based on an existing, previously validated instrument (Parks, Activity and Recreation Among Kids Tool [49], Environmental Assessment for Public Recreation Spaces instrument [50]), while all three included some measure of inter-rater reliability [34,46,47]. GIS studies often defined buffers to identify geographical areas of influence or exposure relative to the participant’s home address (*n* = 6), school neighbourhood (*n* = 8), or residential neighbourhood (using postal code) (*n* = 5). Buffers were estimated using street or walking networks (*n* = 7), radial distance (i.e., Euclidean) (*n* = 9), and administrative boundaries [25]; one study [46] included network and radial buffers. Buffer sizes ranged from 500 [34] to 5000 m [40,41].

The 11 studies that did not use GIS relied on a variety of measures to retrieve urban form data. Three studies used police traffic collision report data [31,32,38] and one study used child death review unit data [26]. Four studies used previously validated aggregate measures such as the Canadian urban sprawl index [42], a walk index [27], Street Smart Walk Score [51], or a neighbourhood design classification [28]. The remaining studies used community-level census data [34], Normalized Difference Vegetation Index for greenness [52], telephone book listings [53], or in-person audits using a previously validated instrument [36].

Food environment was the most frequent urban form measure in the studies reviewed and included food environment within the school (*n* = 5) and residential neighbourhoods (*n* = 6), with one study [46] measuring the food environment in both. Food environment was generally operationalized as proximity to, or density of, various food types. All food environment studies included a measure of fast food restaurants, although they captured these data in different ways. The most common measure of fast food was to indicate the number/density of fast food restaurants within a buffer, [23,26,35,36,37,43,44,45,46], while two studies measured distance to the nearest food outlet of interest [23,26]. Classification of food types varied as two studies scored food options as “healthy” [23,43], with one study [23] considering price as well. The scoring of healthy versus unhealthy food was informed by Canada’s Food Guide [43] or through a previously validated tool [54,55] to classify food quality and price [23]. While not all studies classified the food environment into healthy or unhealthy, they tended to have a mix of food options with only one [53] focussing exclusively on fast food. The remaining studies also considered convenience stores [35,36,37,44,45,46], grocery stores or supermarkets [23,43], and doughnut shops, coffee shops, or bakeries [26,44,45].

### 3.3. Measurement of Health Outcomes

Health outcome data were measured or estimated through researcher or clinician measures (*n* = 13), self-report data (*n* = 6), derived from databases (*n* = 10), or measured with a questionnaire (*n* = 3). Height and weight were primarily measured by trained researchers or at a clinic [23,24,26,36,37,43,44,45,48,56]; however, height and weight were also self-reported [46], parent-reported [47], and reported through the Canadian Community Health Survey [43] or the Health Behaviour in School-Aged Children Survey [26]. Body composition was determined using dual-energy X-ray absorptiometry in two studies [34,35]. Injuries were measured through police traffic collision report data [29,32,33,39,57], health record data [37,57], or insurance reports [25]. Self-reported injuries in the last 12 months were measured in two studies using the Health Behaviour in School-Aged Children Survey [39,41]. Respiratory symptoms or illness were measured through health record data [58]. Allergy symptoms were assessed in one study through physician diagnosis and skin prick testing [52]. Two studies used validated questionnaires to evaluate health outcomes: the Cantril Ladder [28] and the PedsQL questionnaire [58] were used to measure emotional well-being and health-related quality of life, respectively.

### 3.4. Data Mapping

Individual study findings were mapped according to the urban form characteristics and health outcomes examined. A summary of these findings is provided in Table S1, with those studies reporting statistical associations in bold. Additionally, the main findings for each study, along with the direction of the effect (when appropriate), are provided in each cell; that is, when statistically significant results were reported, we indicate a positive (when exposure increases, so too does outcome) or negative relationship (the inverse). If a significant result was observed, but a comparison of categories was performed (i.e., one park type versus another park type), the direction is not reported. If statistical significance was not identified in a study, the direction of the effect is not provided. The most frequent category mapped included studies that examined the relationship between weight status and neighbourhood food environment (*n* = 6). Of these six, five studies indicated a null relationship between weight status and fast food restaurants in the residential neighbourhood food environment [26,36,37,40,43], with one indicating that, using previously developed scoring tools [54,55], healthier fast food restaurants at lower prices were associated with lower BMI [23]. Two studies indicated lower BMI with increased density of convenience stores [23,36]. Density of healthy food options was associated with lower odds of higher BMI [23,43] and greater distance to supermarkets was associated with higher BMI [43]. School neighbourhood food environment results were less consistent; one study indicated an increased chance of high BMI with increased fast food density [46], one indicated lower odds of high BMI with increased density of fast food [53], and three studies had null results [35,44,45]. One study [53] indicated lower odds of BMI with increased number of food retailers near schools regardless of type (fast food, sub/sandwich shop, doughnut shop, etc.). Two studies [44,45] considered bakeries, convenience stores, and grocery stores (in addition to fast food), and both indicated no evidence of a relationship between the density of these food destinations and BMI.

The association between weight status and parks/natural space/greenness was considered in five studies [26,34,37,47,56]. Two of these studies found a statistically significant relationship: one indicated that larger esthetic parks without sport features were associated with lower truncal fat [34] and another indicated that, while park density and distance were not associated with weight status, children had higher odds of a healthy BMI when a playground was within one kilometer of their home [47]. In addition, one study, using recursive partitioning, demonstrated that parks within 500 m of the home may be important for weight status in those at risk of obesity [37].

The relationship between injuries and road/intersection characteristics was the next most frequent relationship investigated (*n* = 5). All five studies included motor vehicle collisions as the primary mechanism of injury; three studies focussed on pedestrians [32,33,38], one focussed on bicyclists [56], and one included pedestrians, bicyclists, and motor vehicle occupants [57]. These studies were diverse in the urban form characteristics examined. For pedestrians, uncontrolled mid-block collisions had higher odds of injury than signalized intersections [32], presence of an arterial road at an intersection was associated with higher odds of injury [57], and road type was not associated with injury severity [33], but in one study, one-way roads had higher odds of injury [38]. Bicyclists had lower odds of injury on roads that were divided with no barrier and demonstrated no association between traffic control devices and injury severity [31].

All but one study [30] provided a statistical analysis where confounders or covariates were considered. Common covariates for studies included age (*n* = 17), sex/gender (*n* = 19), and a measure of socioeconomic status (*n* = 22). Other covariates that were considered included parental BMI [35,36,37,47], diet [23,37,48], physical activity [27,45,53], and peer influence [39,45].

## 4. Discussion

In the Canadian context, associations have been explored between urban form and asthma [27], allergic rhinitis [52], health-related quality of life [58], injury [25,28,29,31,32,33,38,39,41,57], mental health [40,51], and weight status [23,24,26,34,36,37,43,46,47,48,53], for children and adolescents. These health outcomes could have a profound effect on individuals throughout life given that health outcomes experienced in childhood may track into and persist in adulthood [5,6,7]. Even the few health outcomes that were included in our review findings may be of great public health importance given the relationships between urban form and poor health outcomes at such early ages in Canada.

In addition to injury and weight status, previous work focussing on adults indicated associations between urban form and blood pressure, metabolic syndrome, diabetes, mental health, and cardiovascular diseases [4]. Through this review, we identify fewer health outcomes studied in children compared with adults in relation to Canadian urban form [4]. In Canadian adults, land use patterns, urban design features, and transportation systems were important urban form correlates of health [4]. In Canadian children, access to parks/greenspace, road connectivity, and road characteristics appear to be particularly important urban form characteristics associated with health. Additionally, school and home neighbourhood food environments were examined frequently (*n* = 11) with regard to weight status in children, but results were mixed. The evidence gathered in this review seems to indicate that healthier food environments as well as proximity to supermarkets or convenience stores may be associated with a lower BMI in children. In contrast, unhealthy food environments appeared to play less of a role in affecting weight status with the majority of these studies indicating no evidence of a relationship between density of and proximity to fast food restaurants with weight status. It is possible that parents and caregivers are gatekeepers to accessing fast foods, so their presence or proximity may have less impact on the health of younger children [15].

The “Social determinants of health and environmental health promotion” provided a framework specific to the Canadian context [22] that informed the urban form categories used to synthesize evidence included in our review. Within this framework, we note poor representation of studies that included land use mix, zoning regulations, and population or residential density. In addition to the health outcomes identified in the current review, the social determinants of health and environmental health promotion framework includes cardiovascular disease, diabetes, cancers, and infectious disease [22]; again, biomarkers in youth leading to chronic disease could be potential directions for future research. The framework [22] includes not only urban form characteristics and health outcomes but also “fundamental”, “proximate”, and other “intermediate” factors. We restricted the scoping review to Canadian research based on the notion that the Canadian context is fundamentally different, socially, politically, and historically, than countries or regions that an international review may be weighted towards. There remain, however, characteristics (e.g., inequality) related to urban form and health outcomes that should be considered in future reviews.

This scoping review provides important evidence about the current state of knowledge in the Canadian context in regard to the associations between urban form and health outcomes in children and youth. Our scoping review, however, has several limitations. An important consideration for this review was its focus on health outcomes and not health behaviours. While our findings suggest particular associations between urban form and health outcomes in children exist, our review does not provide any evidence about the pathways by which urban form influences health (e.g., via active transportation or diet or collisions). The examination of urban form and health behaviours perhaps warrants a distinct review. While we deliberately excluded studies from our review that focussed on respiratory health and potential correlates (e.g., pollution), we also acknowledge the importance of urban form impacts on respiratory health in children [59,60]. Our exclusion of studies in rural settings means our review findings are generalizable to Canadian urban areas only.

While not a limitation of the review per se, much of the current evidence is based on studies predominantly undertaken in three provinces (Ontario, Alberta, or Quebec). Additional nation-wide data and studies conducted in other provinces and territories would result in a more comprehensive and generalizable evidence base on the relations between urban form and health outcomes in Canadian youth. Moreover, most studies included in the review were published more than 5 years ago which suggests there is a need for newer evidence. The studies reviewed were largely cross-sectional or ecological, making it difficult to draw conclusions regarding causality. This gap presents an opportunity for future research investigating longitudinal relationships between urban form and health outcomes in children and adolescents in Canada. Notably, more robust scientific evidence is needed to inform policy related to urban form that can influence health outcomes in youth.

## 5. Conclusions

Population health interventions have the potential to promote behavioural changes and, ultimately, better health outcomes in society. Modifying the urban form is a key population-level intervention that may influence health outcomes in children and youth. In Canada, there is already evidence exploring the relations between urban form and health outcomes in children and adolescents. In particular, we noted associations between access to parks/greenspace, road connectivity, and road characteristics and weight status and injury. Weight status–neighbourhood food environment was the most frequently studied relationship, perhaps indicating the importance of weight status on health in children and the potential influence on future chronic disease. Given that the urban form is modifiable but semi-permanent, policymakers and urban planners have a responsibility to design urban environments that promote health and reduce the risk of disease and injury among Canadian youth.

## Figures and Tables

**Table 1:** Study count by urban form and health outcomes in Canadian studies.

Measure	Access to Recreation Opportunities	Aggregate Measures of Neighbourhood Design	Residential Neighbourhood Food Environment	Parks/Natural Space/Greenness	Population or Dwelling Density	Road Density	Road or Intersection Characteristics	School Neighbourhood Food Environment	Street Connectivity	Total
Allergic Rhinitis and Sensitization	0	0	0	1	0	0	0	0	0	1
Asthma or Respiratory Illness	0	1	0	0	0	0	0	0	0	1
Emotional Well-Being	0	0	0	2	0	0	0	0	0	2
Health-Related Quality of Life	0	0	0	1	0	0	0	0	0	1
Injury	0	1	0	1	2	1	5	0	0	10
Mortality	0	0	0	0	0	0	1	0	0	1
Weight Status	4	4	6	5	0	0	1	5	1	26
Total	4	6	6	11	2	1	7	5	1	−

## Supplementary Materials

The following are available online at https://www.mdpi.com/article/10.3390/ijerph18084180/s1, Figure S1: MEDLINE search strategy, Table S1: Association between urban form and health outcomes in Canadian studies, 1992–2019.
